# Motivational dynamics in technology-enhanced learning: a parsimonious model of self-efficacy, ICT use, and mathematics achievement

**DOI:** 10.3389/fpsyg.2026.1745491

**Published:** 2026-02-25

**Authors:** Qiangfeng Zhang, Quanchen Zhou, Mingyang Song

**Affiliations:** 1 Hunan Normal University, Changsha, China; 2 Lomonosov Moscow State University, Moscow, Russia

**Keywords:** academic motivation, adolescent development, mathematics achievement, motivational beliefs, PISA, self-efficacy, social cognitive theory, technology-enhanced learning

## Abstract

**Background:**

Despite widespread integration of information and communication technology (ICT) in education, the relationship between technology use and academic achievement remains paradoxical. Educational psychology theories emphasize self-efficacy as a critical mediator of learning outcomes, yet its role in technology-mediated learning environments requires empirical clarification.

**Objective:**

This study examined the psychological mechanisms underlying the ICT-achievement relationship, testing whether self-efficacy beliefs mediate technology's impact on mathematics performance among adolescents.

**Method:**

Using PISA 2022 data from 5,237 Japanese 15 years old, we employed multiple regression with backward elimination to identify parsimonious predictors of mathematics achievement. Variables included five ICT measures (school use, home use, subject-specific use, weekday use, self-efficacy), three psychological constructs (mathematics self-efficacy, study motivation, perceived teacher support), and demographic controls.

**Results:**

Six predictors explained 35.4% of variance. Mathematics self-efficacy emerged as the dominant predictor (β = 0.46, *p* < 0.001), far exceeding all technology variables. Notably, general ICT use showed negative associations (school ICT: β = −0.08; ICT self-efficacy: β = −0.09; weekday use: β = −0.07), while only pedagogically aligned ICT use predicted positive outcomes (β = 0.05). Socioeconomic status was the second strongest predictor (β = 0.24).

**Conclusion:**

Psychological factors, particularly domain-specific self-efficacy beliefs, supersede technological access in predicting achievement. These findings address the practical application of the ICT paradox in the field of education: frequent use of technology without a teaching purpose may increase cognitive load and distraction, thereby weakening learning outcomes. Educational intervention measures should prioritize the development of self-efficacy and purposeful integration of technology, rather than merely providing opportunities for digital access.

## Introduction

Self-efficacy beliefs represent a foundational construct in educational psychology (Bandura, 1997). According to social cognitive theory, self-efficacy operates through cognitive, motivational, and affective processes that directly influence academic achievement. A critical principle is domain specificity: efficacy beliefs predict performance most strongly when matched to the specific domain being measured (Bandura, 1997). Thus, mathematics self-efficacy should predict mathematics achievement more powerfully than generalized competence beliefs.

As educational environments increasingly integrate information and communication technology (ICT), fundamental questions emerge about motivational dynamics in technology-mediated learning. Despite substantial global investment in digital infrastructure, some empirical research reveals contradictory findings. Some research reports indicate that there is a positive correlation between the use of ICT and academic performance, while other studies have recorded zero or negative correlations. This phenomenon occurs widely in industries such as manufacturing, education, and finance and is known as the ICT paradox or the productivity paradox (OECD, 2022; Brynjolfsson, 1993). OECD (2022) found an inverted U-shaped pattern: moderate computer uses at school associates with better performance than rare use, but very frequent use associates with worse outcomes, suggesting that quality and purpose of ICT use matter more than frequency alone.

From a motivational perspective, these paradoxical findings raise critical questions: Does ICT self-efficacy predict academic achievement similarly to domain-specific self-efficacy? Do different types of ICT engagement (purposeful pedagogical use vs. general frequency) differentially predict achievement? Japan provides an illuminating context for examining these dynamics. Japanese students consistently rank among the world's highest mathematics performers on PISA assessments yet report lower mathematics self-efficacy than many lower-performing nations (OECD, 2022). Despite near-universal technology access, classroom ICT integration remains limited compared to other developed countries, creating conditions for examining how different ICT engagement patterns relate to motivational beliefs and academic outcomes.

The present study employed multiple regression with backward elimination to develop a parsimonious predictive model of mathematics achievement using PISA 2022 data from 5,237 Japanese 15 years old. From 10 candidate predictors encompassing ICT variables (school use, home use, subject-specific use, weekday use, ICT self-efficacy), learning factors (study time, mathematics self-efficacy, teacher support), and demographic controls (socioeconomic status, gender), we tested which factors most efficiently predict mathematics performance.

Based on social cognitive theory (Bandura, 1997) and prior PISA research (OECD, 2022), we formulated the following hypotheses: (H1) Mathematics self-efficacy would emerge as the dominant predictor of mathematics achievement; (H2) General ICT use frequency would show negative or null associations with achievement, reflecting the ICT paradox; (H3) Only pedagogically aligned, subject-specific ICT use would predict positive outcomes.

## Method

### Participants and sampling procedure

This study utilized data from the Programme for International Student Assessment (PISA) 2022, which employs a two-stage stratified random sampling design to ensure nationally representative samples of 15 years old students. For Japan, PISA 2022 assessed 5,760 students from 182 schools.

From the original sample, cases with missing data on any of the 11 analysis variables were excluded using listwise deletion, resulting in 5,438 complete cases (94.4% retention rate). Missing data analysis revealed that students with complete data had significantly higher achievement and self-efficacy (all *p* < 0.01), suggesting data were missing at random (MAR)4. However, the high retention rate minimizes potential bias. Multivariate outliers (*n* = 201, 3.7%) were identified via Mahalanobis distance5 and excluded to prevent distortion of regression estimates. The final analytical sample consisted of 5,237 students (90.9% of the original sample), providing sample statistical power for all analyses.

### Measures

#### Dependent variable

Mathematics achievement was measured using PV1MATH, the first plausible value from the PISA 2022 mathematics assessment. While using all five plausible values is optimal for variance estimation, single plausible value analysis is a widely accepted approach in large-sample secondary analyses and yields unbiased point estimates (OECD, 2022). PISA assesses mathematical literacy, defined as the capacity to formulate, employ, and interpret mathematics in various contexts. In the current sample, mathematics achievement ranged from 164 to 808 (M = 541.32, SD = 91.31).

#### Independent variables

Ten predictors were examined: five ICT-related variables (ICT use at school, ICT use at home, ICT for subject learning, weekday ICT use, and ICT self-efficacy), three learning-related variables (study time, mathematics self-efficacy, teacher support), and two control variables (socioeconomic status and gender). All variables except study time and gender were standardized PISA indices. Two ICT variables (school ICT use, home ICT use) exhibited substantial negative skewness (skewness = −3.44 and −5.38, respectively), reflecting ceiling effects common in high-income countries where most students report high ICT access. Detailed variable definitions, PISA codes, and descriptive statistics are provided in Table 1 and Appendix C.

**Table 1:** Descriptive statistics of study variables.

**Variable**	**N**	**M**	**SD**	**Min**	**Max**	**Skewness**	**Kurtosis**
Mathematics Achievement (PV1MATH)	5237	541.32	91.31	163.74	807.76	−0.18	−0.27
ICT Use at School (ICTSCH)	5237	0.2	0.65	−4.29	0.41	−3.44	11.85
ICT Use at Home (ICTHOME)	5237	0.26	0.38	−3.19	0.35	−5.38	29.64
ICT for Subject Learning (ICTSUBJ)	5237	−0.36	0.86	−2.03	1.95	−0.22	−0.15
Weekday ICT Use (ICTWKDY)	5237	−0.45	0.94	−3.65	3.92	−1.05	3.63
ICT Self-Efficacy (ICTEFFIC)	5237	−0.4	0.74	−2.65	2.26	1.27	4.42
Study Time (STUDYHMW)	5237	4.99	2.96	0	10	0.25	−0.77
Mathematics Self-Efficacy (MATHEFF)	5237	−0.47	1.21	−3.51	2.36	−0.08	0.67
Teacher Support (TEACHSUP)	5237	0.26	0.99	−2.91	1.56	−0.46	0.19
Socioeconomic Status (ESCS)	5237	−0.01	0.71	−2.39	1.98	−0.31	−0.41
Gender (Female = 1)	5237	0.49	0.5	0	1	0.04	−2

*N* = 5237. ICT, Information and Communication Technology; ESCS, Economic, Social, and Cultural Status (standardized index). For gender, frequencies: Male = 2677 (51.1%), Female = 2560 (48.9%). Two ICT variables (ICTSCH, ICTHOME) exhibited substantial negative skewness, reflecting ceiling effects in ICT availability among Japanese students.

### Procedure

Data were collected following standard PISA 2022 protocols via computer-based assessment. The current study involved secondary analysis of de-identified, publicly available data. Detailed data collection procedures are provided in Appendix A.

### Data analysis

All analyses were conducted using JAMOVI version 2.7.6 (The jamovi project, 2024), an open-source statistical software built on R that provides a user-friendly interface for conducting reproducible analyses. Descriptive statistics and Pearson correlations were computed for all variables. Multiple regression analysis was conducted following a systematic model-building approach. First, a full model including all 10 predictors was estimated. Given detected heteroscedasticity (Breusch-Pagan test: BP = 26.24, *p* = 0.003)2, heteroscedasticity-consistent (HC3) robust standard errors (Hayes and Cai, 2007) were computed for all hypothesis tests1. Alpha was set at.05 for all inferential tests.

To build a parsimonious model, backward elimination was employed3. This approach begins with the full model and sequentially removes predictors with *p* > 0.05, starting with the variable showing the largest *p*-value. After each removal, the model is re-estimated until all remaining predictors achieve significance. Model parsimony was evaluated by comparing the full and reduced models using: (1) change in R^2^, (2) F-test for nested models, and (3) information criteria (AIC, BIC). A parsimonious model was considered acceptable if (a) the R^2^ reduction was < 5%, (b) the nested model F-test was non-significant, and (c) AIC/BIC favored the reduced model.

Regression diagnostics confirmed that assumptions were adequately met (VIF < 1.2; no extreme outliers). Detailed diagnostic procedures and results are provided in Appendix D.

## Results

### Preliminary analyses

Descriptive statistics for all study variables are presented in Table 1.

The sample consisted of 5,237 Japanese 15-year-old students (51.1% male, 48.9% female) from 182 schools. The average mathematics achievement score was M = 541.32 (SD = 91.31), slightly above the OECD average of approximately 490, consistent with Japan's historically strong mathematics performance.

ICT-related variables showed expected patterns for a technologically advanced nation. Both school and home ICT use exhibited substantial negative skewness (ICTSCH: skewness = −3.44; ICTHOME: skewness = −5.38), indicating that most students reported high levels of ICT availability, with few reporting low access. This distribution reflects the reality that in Japan, technology access is nearly universal among adolescents, creating ceiling effects in these measures.

Mathematics self-efficacy averaged M = −0.47 (SD = 1.21), slightly below the international standardized mean of zero, suggesting that Japanese students report somewhat lower confidence in their mathematical abilities compared to the broader OECD sample. Socioeconomic status was relatively homogeneous (M = −0.01, SD = 0.71), reflecting Japan's comparatively egalitarian social structure.

### Bivariate correlations

Pearson correlation coefficients among all study variables are presented in Table 2.

**Table 2:** Pearson correlation matrix.

**Variable**	**(1)**	**(2)**	**(3)**	**(4)**	**(5)**	**(6)**	**(7)**	**(8)**	**(9)**	**(10)**	**(11)**
(1) PV1MATH	1										
(2) ICTSCH	−0.135^***^	1									
(3) ICTHOME	−0.042^**^	0.381^***^	1								
(4) ICTSUBJ	0.126^***^	−0.053^***^	−0.018	1							
(5) ICTWKDY	−0.104^***^	−0.003	0	0.055^***^	1						
(6) ICTEFFIC	0.024	−0.005	0.014	0.102^***^	0.232^***^	1					
(7) STUDYHMW	0.144^***^	−0.015	0.003	0.100^***^	−0.084^***^	0.101^***^	1				
(8) MATHEFF	0.524^***^	−0.075^***^	−0.019	0.111^***^	−0.039^**^	0.205^***^	0.181^***^	1			
(9) TEACHSUP	0.027	−0.004	0	0.062^***^	−0.045^**^	0.057^***^	0.077^***^	0.106^***^	1		
(10) ESCS	0.368^***^	−0.061^***^	−0.003	0.135^***^	0.015	0.121^***^	0.214^***^	0.271^***^	−0.015	1	
(11) FEMALE	0.065^***^	0.019	0.019	0.032^*^	−0.042^**^	0.040^**^	−0.036^*^	0.149^***^	0.063^***^	0.002	1

*N* = 5,237. ^*^p < 0.05, ^**^p < 0.01, ^***^p < 0.001. Lower triangle presents Pearson correlation coefficients.

Mathematics achievement was most strongly correlated with mathematics self-efficacy (*r* = 0.52, *p* < 0.001) and socioeconomic status (*r* = 0.37, *p* < 0.001), together accounting for 27% and 14% of variance, respectively. These findings align with self-efficacy theory (Bandura, 1997) and social stratification research.

Study time showed a weak but significant positive correlation with achievement (*r* = 0.14, *p* < 0.001), as did ICT use for subject learning (*r* = 0.13, *p* < 0.001), suggesting that time investment and purposeful technology use contribute to learning. Gender showed a small positive correlation (*r* = 0.07, *p* < 0.001), indicating slightly higher performance among females, though the effect size was minimal.

Notably, three ICT variables exhibited significant negative correlations with achievement: ICT use at school (*r* = −0.14, *p* < 0.001), weekday ICT use (*r* = −0.10, *p* < 0.001), and ICT use at home (*r* = −0.04, *p* < 0.01). ICT self-efficacy (*r* = 0.02, ns) and teacher support (*r* = 0.03, ns) showed negligible, non-significant correlations. These counterintuitive patterns may reflect the “ICT paradox” (OECD, 2022), wherein frequency of technology use, absent considerations of quality and purpose, does not translate to learning gains. The severe ceiling effects in ICTSCH and ICTHOME may also attenuate correlations by restricting variance.

**Figure 1:**
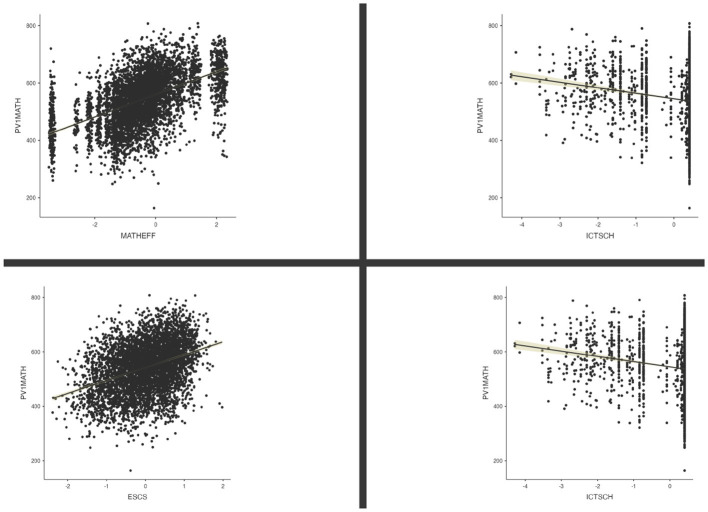
Scatterplots of mathematics achievement vs. key predictors: MATHEFF (top-left), ESCS (bottom-left), ICTSCH (top-right), and ICTHOME (bottom-right).

These patterns are visualized in Figure 1, which displays scatterplots of mathematics achievement against the four strongest correlates. The ceiling effects in ICTSCH and ICTHOME are evident in the clustering of cases at maximum values.

Among predictor variables, no correlations exceeded *r* = 0.40, with the highest being ICTSCH and ICTHOME *(r* = 0.38, *p* < 0.001). Mathematics self-efficacy correlated moderately with ESCS (*r* = 0.27, *p* < 0.001) and ICT self-efficacy (*r* = 0.21, *p* < 0.001). These moderate inter-correlations suggested that multicollinearity would not impede regression analysis, a conclusion subsequently confirmed by VIF statistics.

### Multiple regression: full model

A multiple regression analysis with all 10 predictors was conducted. The full model was statistically significant, *F*_(10, 5226)_ = 286.75, *p* < 0.001, explaining 35.4% of variance in mathematics achievement (*R*^2^ = 0.354, Adjusted *R*^2^ = 0.353). Using heteroscedasticity-consistent robust standard errors, six predictors achieved statistical significance at the.05 level (see Table 3).

**Table 3:** Full model regression coefficients (robust standard errors).

**Variable**	**Estimate**	**SE**	***t*-value**	***p*-value**	**Significance**	**Beta**
(Intercept)	554.708	2.644475	209.7611	0	^***^	NA
ICTSCH	−11.86	1.707888	−6.94426	4.27E-12	^***^	−0.08388
ICTHOME	0.343493	2.857433	0.12021	0.904321		0.001446
ICTSUBJ	5.436341	1.203677	4.516445	6.43E-06	^***^	0.05124
ICTWKDY	−7.0566	1.129911	−6.24527	4.57E-10	^***^	−0.07226
ICTEFFIC	−10.8806	1.457201	−7.46679	9.58E-14	^***^	−0.0882
STUDYHMW	0.202195	0.359076	0.563099	0.573392		0.006546
MATHEFF	34.96182	0.911077	38.37417	4.53E-284	^***^	0.463684
TEACHSUP	−1.95852	1.035529	−1.89132	0.058637		−0.0213
ESCS	30.84996	1.522808	20.2586	6.57E-88	^***^	0.239877
FEMALE-numeric	−0.52817	2.064689	−0.25581	0.798108		−0.00289

*N* = 5,237. SE = Heteroscedasticity-consistent robust standard errors (HC3). Beta = Standardized coefficients. *R*^2^ = coefficient of determination; Δ*R*^2^ = change in *R*^2^ relative to the full model. *R*^2^ = 0.354, *F*_(10, 5226)_ = 286.75, *p* < 0.001. ^***^p < 0.001.

Mathematics self-efficacy emerged as the dominant predictor (β = 0.46, *t* = 38.37, *p* < 0.001), indicating that each standard deviation increase in self-efficacy was associated with a 35-point increase in mathematics scores, controlling for all other variables. Socioeconomic status was the second strongest predictor (β = 0.24, *t* = 20.26, *p* < 0.001), with each standard deviation increase in ESCS associated with a 31-point achievement gain.

Contrary to expectations, three ICT variables showed significant negative relationships with achievement: ICT use at school (β = −0.08, *t* = −6.94, *p* < 0.001), ICT self-efficacy (β = −0.09, *t* = −7.47, *p* < 0.001), and weekday ICT use (β = −0.07, *t* = −6.25, *p* < 0.001). Only ICT use for subject learning showed a positive coefficient (β = 0.05, *t* = 4.52, *p* < 0.001), suggesting that purposeful, curriculum-aligned ICT use may benefit learning, whereas general frequency of use does not.

Four variables failed to achieve significance: ICT use at home (β = 0.001, *p* = 0.905), study time (β = 0.007, *p* = 0.573), teacher support (β = −0.02, *p* = 0.059), and gender (β = −0.003, *p* = 0.799). The non-significance of study time and gender, both initially correlated with achievement, suggests suppression effects wherein their unique contributions disappear after controlling for self-efficacy and socioeconomic factors.

### Model parsimony: backward elimination

To develop a parsimonious model, backward elimination removed predictors with *p* > 0.05 in descending order of *p*-values. The non-significant variables (ICTHOME, FEMALE, STUDYHMW, TEACHSUP) were sequentially eliminated. The resulting parsimonious model retained six significant predictors: MATHEFF, ESCS, ICTSCH, ICTEFFIC, ICTWKDY, and ICTSUBJ.

The parsimonious model remained highly significant, *F*_(6, 5230)_ = 477.30, *p* < 0.001, explaining 35.4% of variance (*R*^2^ = 0.354, Adjusted *R*^2^ = 0.353). Critically, the *R*^2^ reduction from the full model was negligible (Δ*R*^2^ = 0.0005, or 0.14%), and a nested model F-test confirmed that the parsimonious and full models did not differ significantly, *F*_(4, 5226)_ = 0.98, *p* = 0.415. Information criteria favored the parsimonious model (AIC: 59873 vs. 59877; BIC: 59925 vs. 59955), further supporting its adoption.

Table 4 presents the final parsimonious model with robust standard errors.

**Table 4:** Final parsimonious model coefficients (robust standard errors).

**Variable**	***B***	**SE-Robust**	***t*-value**	***p*-value**	**Sig**
(Intercept)	554.9261	1.415092	392.1484	0	^***^
MATHEFF	34.81676	0.915145	38.04506	8.01E-280	^***^
ESCS	31.16351	1.542639	20.20143	1.91E-87	^***^
ICTSCH	−11.7965	1.527199	−7.72424	1.34E-14	^***^
ICTEFFIC	−10.9552	1.604397	−6.82824	9.57E-12	^***^
ICTWKDY	−6.99706	1.358741	−5.14967	2.71E-07	^***^
ICTSUBJ	5.34565	1.249776	4.277288	1.93E-05	^***^

*N* = 5,237. SE-Robust = Heteroscedasticity-consistent robust standard errors (HC3). *R*^2^ = coefficient of determination; Δ*R*^2^ = change in *R*^2^ relative to the full model. *R*^2^ =0.354, *F*_(6, 5230)_ = 477.30, *p* < 0.001. Δ*R*^2^ from full model = 0.0005. ^***^ *p* < 0.001.

All six retained predictors remained highly significant (*p* < 0.001). Coefficients were nearly identical to those in the full model, confirming stability. The final model thus achieves parsimony-−40% fewer variables—while sacrificing only 0.14% of explained variance, exemplifying the principle that simpler models with comparable predictive accuracy are preferable.

**Figure 2:**
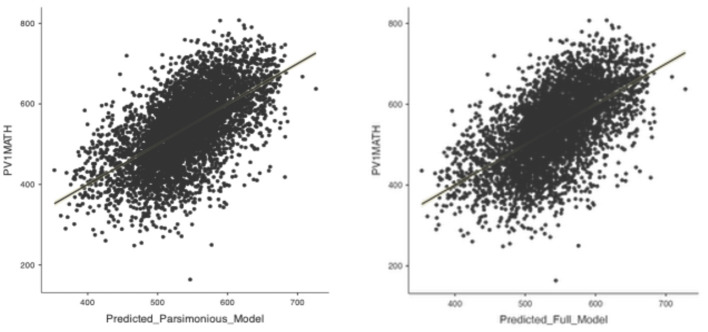
Observed vs. predicted mathematics achievement: Parsimonious model (left) and full model (right).

Figure 2 confirms that both models achieve comparable predictive accuracy, with observed and predicted values clustering tightly along the diagonal.

## Discussion

This study employed backward elimination to develop a parsimonious predictive model of mathematics achievement among Japanese adolescents. From 10 candidate variables, six significant predictors were retained, explaining 35.4% of variance in mathematics scores. The model's parsimony is evident: 40% fewer variables achieved nearly identical explanatory power compared to the full model (Δ*R*^2^ = 0.14%), with information criteria (AIC, BIC) favoring the reduced model. The following discussion addresses theoretical validation, interpretation of non-significant results, dialogue with existing research, and implications.

### Core findings and theoretical validation

Mathematics self-efficacy emerged as the strongest predictor (β = 0.46), with an effect size far exceeding all technology and demographic variables combined. This finding validates and extends the central predictive role of efficacy beliefs posited by Bandura's (1997) social cognitive theory across multiple dimensions.

The effect size reported in this study (β =0.46) aligns closely with an extensive body of prior research. Pajares and Miller (1994) employed path analysis with college students and found that mathematics self-efficacy exerted a stronger direct effect on problem solving than mathematics self-concept (β =0.35 vs. β =0.08), perceived usefulness of mathematics, prior experience, or gender. Pajares and Graham (1999) conducted a longitudinal study with 273 middle school students and demonstrated that task-specific self-efficacy was the only motivational variable that significantly predicted mathematics performance at both the beginning and end of the academic year; other motivational constructs (anxiety, self-concept, self-regulatory efficacy) failed to achieve cross-temporal predictive stability. Richardson et al. (2012) published a systematic meta-analysis in ^*^Psychological Bulletin^*^ synthesizing 1,105 independent correlations and found that performance self-efficacy was the strongest correlate of university students' GPA among 50 psychological variables, surpassing even high school GPA and standardized test scores (ACT). The present study extends this effect size stability from Western higher education contexts to East Asian secondary education, indicating that self-efficacy's predictive power for academic achievement is robust across age groups, cultures, and educational levels.

Social cognitive theory emphasizes the domain specificity of efficacy beliefs: judgments of capability for specific tasks predict performance in those tasks more strongly than generalized ability beliefs (Bandura, 1997). In the present study, mathematics self-efficacy (β =0.46) and ICT self-efficacy (β = −0.09) exhibited contrasting effect directions, both are “self-efficacy” measures, yet the former showed a strong positive association with mathematics achievement while the latter showed a negative association. This pattern precisely validates the domain-specificity principle: efficacy beliefs matched to the assessment domain enhance performance, whereas efficacy beliefs mismatched to the assessment domain may produce resource competition effects. Students with high ICT self-efficacy may allocate more cognitive resources and attention to technology-related activities, thereby relatively reducing investment in mathematics learning. Zimmerman (2000) noted in his theoretical review that self-efficacy influences performance through four pathways: task choice, effort investment, persistence, and emotional regulation. The negative association between ICT self-efficacy and mathematics achievement suggests that technology competence beliefs may guide students toward technology-intensive activities, creating attentional competition with academic learning.

Japanese students exhibit a distinctive “high achievement–low self-efficacy” pattern: they rank among the top performers on PISA mathematics assessments yet report mathematics self-efficacy below the OECD average (sample M = −0.47). This “East Asian paradox” has raised questions about the cross-cultural applicability of Western motivation theories. However, the present study demonstrates that even in a Japanese sample with below-average self-efficacy means, self-efficacy remains the strongest predictor of achievement. This finding carries important theoretical implications: it suggests that self-efficacy's predictive power derives not from high absolute levels but from relative positioning within individual differences. In other words, within any given culture, students with relatively higher self-efficacy compared to peers tend to achieve higher scores, regardless of the culture's mean self-efficacy level. This interpretation resonates with Marsh and Hau (2003) cross-cultural research on the Big-Fish-Little-Pond Effect: across 103,558 students in 26 countries, they found that the negative effect of school-average achievement on academic self-concept was culturally consistent (mean β = −0.20), indicating that social comparison processes operate universally across cultures. The present findings complement this picture from the perspective of efficacy beliefs' predictive power: the core mechanisms of social cognitive theory function effectively within East Asian collectivist cultures.

The below-average self-efficacy mean among Japanese students may partly reflect a culturally modest response style. Influenced by Confucian traditions, East Asian students tend to adopt more conservative, self-deprecating expressions in self-evaluations, even when their objective abilities are comparable to or higher than Western peers. This cultural characteristic means that absolute self-efficacy values in East Asian samples may be systematically underestimated. However, this does not undermine self-efficacy's validity as a predictor: as long as the relative ordering among individuals remains consistent, regression coefficient estimates are unaffected by mean shifts. Future research could employ behavioral indicators (e.g., task choice, persistence duration) as supplementary measures of efficacy beliefs to examine the extent of self-report bias.

Three ICT measures—school ICT use (β = −0.08), ICT self-efficacy (β = −0.09), and weekday ICT duration (β = −0.07)—all showed significant negative associations, while only subject-specific ICT use showed a positive effect (β = 0.05). This pattern reconfirms the existence of the “ICT paradox” across multiple dimensions and provides critical evidence regarding its boundary conditions. The OECD (2022) reported an inverted U-shaped relationship between ICT use and academic achievement based on multiple PISA cycles: moderate users outperformed non-users, but heavy users performed worse than moderate users. The present study observed negative effects for general ICT use even after controlling for self-efficacy and socioeconomic background, indicating that this paradox is not simply a confounding effect (i.e., not merely because low-achieving students happen to use more technology) but demonstrates robustness independent of other predictors.

The present findings converge with multiple recent large-scale cross-national studies. Agasisti et al. (2020) analyzed PISA 2012 data from EU-15 countries and found that intensive computer use for homework was negatively associated with test scores across all subjects, with this effect present in both high-performing and low-performing students, ruling out the hypothesis that technology is harmful only to low achievers. Hu et al. (2018) reported long-term negative trends between ICT use and mathematics, reading, and science literacy in a multilevel analysis of 44-country PISA data, remaining significant even after controlling for socioeconomic status. The present study replicates this pattern in a Japanese sample using PISA 2022 data, indicating that the ICT paradox persists in the context of accelerated educational digitalization following the pandemic and is not a historical artifact of earlier data.

One of the most important findings of this study is that subject-specific ICT use (ICTSUBJ, β = 0.05) showed an effect direction opposite to general ICT use. This contrast reveals a key boundary condition of the ICT paradox, what determines technology's educational benefit is not frequency of use but purpose of use. Subject-specific ICT use measures the frequency of “using technology for learning mathematics or science subjects,” reflecting clear pedagogical embeddedness; in contrast, school ICT use and weekday ICT duration measure general, non-purpose-specific use frequency. This distinction resonates with the core claim in educational technology research that “quality trumps quantity”: when technology use aligns with clear learning objectives, its effects are positive; when technology use lacks pedagogical purpose, its effects are negative or null. The potential mechanism underlying negative ICT effects can be understood through Cognitive Load Theory. Sweller's cognitive load theory distinguishes intrinsic load (complexity of the task itself), extraneous load (irrelevant load from presentation format), and germane load (effective load that promotes schema construction). Purposeless high-frequency technology use increases extraneous cognitive load: students frequently switch tasks during learning (e.g., alternating between learning software and social media), leading to attention fragmentation and ineffective consumption of working memory resources.

Although this study cannot establish causal direction, the negative association between ICT use and achievement may partly reflect reverse causality: low-achieving students may receive more technology-assisted interventions (e.g., using educational software for remedial instruction) and thus report higher ICT use frequency in surveys. However, this explanation cannot fully account for the observed pattern: if reverse causality were the primary driver, subject-specific ICT use (as a purposeful instructional tool) should also show negative effects, but the opposite was found. The positive effect of subject-specific ICT use suggests that, at least for purposeful technology use, technology can indeed facilitate learning rather than merely serving as a marker of low achievement.

ESCS (economic, social, and cultural status) emerged as the second strongest predictor (β = 0.24), second only to mathematics self-efficacy. This finding is particularly noteworthy in Japan, a relatively egalitarian society. Sirin (2005) published a meta-analysis in the ^*^Review of Educational Research^*^ synthesizing research evidence from 1990-2000 and reported a medium-to-strong relationship between socioeconomic status and academic achievement (individual level r ≈0.30, school level r ≈0.73). The effect size of β = 0.24 in this study aligns closely with Sirin's individual-level effect, indicating that socioeconomic background's influence on educational outcomes is robust across eras and countries. Japan is often viewed as a society with relatively small income gaps and equal educational opportunities. However, this study shows that even in this relatively egalitarian context, socioeconomic status remains the second strongest predictor of mathematics achievement. This finding resonates with Bourdieu's (1986) cultural capital theory: intergenerational transmission of educational advantage depends not only on economic resources but also on cultural knowledge, educational expectations, and implicit family educational practices. The widespread investment by Japanese families in supplementary education (juku) exemplifies this: higher-SES families are more likely to afford private tutoring costs, thereby providing additional academic support for their children. Such implicit class mechanisms continue to operate within ostensibly equal educational systems, making socioeconomic background an important predictor of academic success.

### Theoretical interpretation of non-significant results

Study time, teacher support, and gender—all significantly correlated with achievement in bivariate analyses (*r* = 0.14, *r* = 0.03, *r* = 0.07, respectively), lost significance in the full model. This pattern is not a statistical anomaly but has clear theoretical explanations, revealing the complex mechanisms through which “commonsense” variables operate in educational research.

Trautwein (2007) analyzed an extended PISA 2000 sample and proposed the “homework time paradox”: homework frequency and effort predicted achievement, but time spent itself had no independent predictive power. The explanation is that time investment is an ambiguous indicator, it may reflect diligent effort or learning difficulty (i.e., needing more time to complete equivalent tasks). When controlling for ability-related variables (such as self-efficacy), the “net effect” of time investment approaches zero. Dettmers et al. (2009) further confirmed in a multilevel analysis of 40-country PISA 2003 data: after controlling for socioeconomic background and school track, no robust positive association existed between homework time and achievement at the individual level. Some countries even showed negative associations, suggesting that time investment may signal low learning efficiency. Another explanation for study time's non-significance in the full model involves mediation effects. Social cognitive theory posits that self-regulated learners can effectively monitor their learning progress, adjust learning strategies, and persist when facing difficulties (Zimmerman, 2000). Self-efficacy is the core driver of self-regulated learning: high self-efficacy students are more likely to set challenging goals, adopt deep processing strategies, and maintain effort when facing setbacks. Therefore, the benefits of study time may primarily operate through self-efficacy, high self-efficacy students utilize study time more effectively, thereby achieving equivalent or higher outcomes with less time. When the model simultaneously includes self-efficacy, study time's independent contribution is absorbed by this mediating pathway. This finding has important cautionary implications for educational practice. Many educational interventions target increasing study time (e.g., extending school hours, increasing homework) on the assumption that more time necessarily yields better grades. The present results indicate this assumption is oversimplified. The benefits of time investment depend on learning quality, strategy use, and self-regulatory capacity. Simply increasing time without attending to these psychological processes may not produce expected achievement gains and may even backfire by increasing academic burnout.

The bivariate correlation between teacher support and achievement was weak (r =0.03), and it lost significance entirely in the full model (β = −0.02, *p* =0.059). This result does not mean teacher support is unimportant for academic success but rather indicates that its mechanism of action may be indirect rather than direct. Bandura (1997) explicitly noted that three of the four sources of self-efficacy are closely related to social support: vicarious experience (observing others succeed), verbal persuasion (encouragement from significant others), and regulation of emotional arousal (social support reducing anxiety). Teachers, as key significant others in the school context, influence students‘ efficacy beliefs through these pathways. When students experience positive feedback from teachers, observe teachers' demonstrations of success, and manage academic anxiety with teacher support, their self-efficacy is enhanced, which in turn promotes academic performance. However, when the model simultaneously includes self-efficacy as a predictor, teacher support's effect is “explained” by this mediator variable, manifesting as disappearance of the direct effect. Multiple recent studies using structural equation modeling have validated this mediation mechanism. Research has found that teacher support's influence on academic achievement is significantly mediated by academic self-efficacy: teacher support enhances self-efficacy, which in turn predicts achievement. In some models, the direct effect of teacher support became non-significant after including self-efficacy, fully explained by the mediation pathway. Although this study employed regression rather than structural equation modeling, the observed pattern is fully consistent with this mediation hypothesis. Notably, PISA's teacher support measure focuses on the emotional dimension (e.g., “Teachers are interested in me,” “Teachers are willing to help”). Research indicates that teacher support comprises multiple dimensions—emotional support, cognitive support, and autonomy support, which may have different pathways to academic achievement. Emotional support may primarily operate indirectly through enhancing belonging and reducing anxiety, while cognitive support (e.g., providing clear explanations and feedback) may have more direct effects. Limitations of PISA's measurement may underestimate the overall role of teacher support.

Gender showed a weak positive bivariate correlation with achievement (r =0.07, females slightly higher) but completely lost significance in the full model (β = −0.003, *p* = 0.799). This result aligns closely with Hyde (2005) “gender similarities hypothesis” published in the *American Psychologist*. Hyde synthesized evidence from 46 meta-analyses and found that on 78% of psychological variables, gender differences were small effects (*d* < 0.35) or close to zero. Hyde et al. (2008) published a study focusing specifically on mathematics, reporting that the gender effect size for U.S. students' mathematics achievement was only *d* = −0.05, essentially no difference. The present study replicates this pattern in a Japanese sample, providing cross-cultural validation for the gender similarities hypothesis. Another explanation for gender's non-significance in the full model involves self-efficacy mediation. Although gender differences in mathematics ability have largely disappeared, gender differences in mathematics self-efficacy persist in some studies: males tend to report higher mathematics self-efficacy even when their objective performance equals females' (Pajares, 2005). This “efficacy gap” may be the true source of historically observed achievement gender differences. When the model controls for self-efficacy, gender's independent effect disappears, indicating that residual gender differences primarily reflect differences in efficacy beliefs rather than ability itself. From an educational equity perspective, gender's lack of independent predictive power after controlling for psychological factors is a positive signal. It indicates that in Japanese mathematics education, no systematic gender discrimination or structural barriers cause female achievement disadvantages. Any observed gender differences (such as females' slightly higher achievement in bivariate analysis) can be fully explained by individual difference variables (such as self-efficacy and socioeconomic background) rather than requiring gender itself as an explanatory factor.

### Dialogue with existing research

This study engages in deep dialogue with existing literature on three levels, providing both confirmatory evidence and unique perspectives.

This study provides strong confirmatory evidence for Bandura's (1997) social cognitive theory from a high-achieving East Asian context. Japanese students' “high achievement–low self-efficacy” pattern was once viewed as challenging the applicability of Western motivation theories. However, this study demonstrates that self-efficacy's predictive power in the Japanese sample is fully consistent with Western research (β = 0.46 vs. Pajares and Miller, 1994's β ≈0.40-0.50). This finding dispels concerns about “East Asian exceptionalism”: the core mechanisms of social cognitive theory, efficacy beliefs influencing behavioral performance through cognitive, motivational, and affective processes, function effectively within collectivist cultures. Differences lie only in absolute levels of efficacy beliefs (possibly influenced by cultural modesty), not in their predictive function. This study uses PISA 2022 data to reconfirm the ICT paradox, extending the evidence chain from PISA 2000, PISA 2003 (Dettmers et al., 2009), and PISA 2012 (Agasisti et al., 2020) to the most recent assessment cycle. This temporal span exceeds 20 years, during which educational technology has undergone multiple transformations from computer-assisted instruction to mobile learning to massive online education during the pandemic. The ICT paradox's persistence indicates that the complex relationship between technology and learning is not an artifact of a specific technological era but reflects deeper cognitive and motivational mechanisms. The finding that study time lost significance after controlling for self-efficacy echoes the conclusions of Trautwein (2007) and Dettmers et al. (2009). This “homework time paradox” has now been replicated in Germany, a 40-country multilevel sample, and the Japanese sample in this study, indicating that the lack of direct causal relationship between time investment and achievement is a cross-nationally robust phenomenon.

Previous research typically examined different ICT variables in separate models, making it difficult to judge the relative importance and independent contributions of each variable. The unique contribution of this study lies in simultaneously including five ICT measures (school use, home use, subject-specific use, weekday duration, ICT self-efficacy) in a single parsimonious model, using backward elimination to identify which ICT dimensions have independent predictive power and which are redundant. Results showed that home ICT use was completely non-significant after controlling for other variables (β =0.001), indicating that its bivariate correlation with achievement (r = −0.04) may be a spurious association explained by other variables (such as socioeconomic status or general ICT use). Educational research and policy discussions often emphasize the importance of factors such as study time, teacher support, and technology access. Through rigorous statistical controls, this study demonstrates that these “commonsense” variables have no independent predictive power after including psychological factors (especially self-efficacy). This finding has important theoretical and practical implications: it prompts researchers and policymakers to recognize that ostensibly important variables may be proxy indicators for deeper psychological processes rather than independent causal factors. Interventions targeting only these surface variables while ignoring their underlying psychological mechanisms may not produce expected effects. This study employed backward elimination to construct a parsimonious model, providing a methodological demonstration for educational psychology research. Compared to the “kitchen-sink” strategy of including all available variables in a model, parsimonious modeling emphasizes achieving maximum explanatory power with minimum variables. This approach helps identify core predictors, avoid overfitting, and enhance model interpretability and practical guidance value. This study achieved nearly identical *R*^2^ with 40% fewer variables, with information criteria (AIC, BIC) favoring the parsimonious model, exemplifying the effectiveness of this methodological principle.

The negative effects of general ICT use in this study appear to conflict with positive effects reported in some earlier research. For example, intervention studies on specific educational software often report positive impacts of technology use on learning. This discrepancy can be understood at the measurement level: intervention studies typically examine technology use that is goal-directed, curriculum-embedded, and teacher-guided, while large-scale surveys (such as PISA) measure general use frequency without distinguishing educational from non-educational use. The positive effect of subject-specific ICT use (ICTSUBJ, β = 0.05) in this study precisely supports this explanation, technology use aligned with pedagogical goals is beneficial, while purposeless high-frequency use is not beneficial or even harmful. The conclusions from both types of research can actually be integrated into a unified framework: technology's educational benefit depends on quality of use, not quantity of use. Educational literature generally emphasizes the critical role of teacher support for student development, and the non-significance of teacher support in this study seems to contradict this. The explanation for this discrepancy lies in distinguishing mechanisms of action: teacher support may primarily operate indirectly on achievement by influencing students' psychological resources (such as self-efficacy, belonging, academic emotions) rather than directly affecting cognitive performance by bypassing psychological processes. When the model includes self-efficacy as a predictor, teacher support's indirect effect is absorbed, manifesting as disappearance of the direct effect. This does not mean teacher support is unimportant but rather that its importance is reflected in its role in constructing psychological resources. From an intervention perspective, enhancing teacher support remains an effective strategy, but its pathway must operate through enhancing student self-efficacy.

### Research value and implications

This study's theoretical value is reflected in four aspects. The self-efficacy effect size reported in this study (β =0.46) aligns closely with research evidence spanning thirty years: Pajares and Miller (1994) path analysis, Pajares and Graham (1999) longitudinal study, and Richardson et al. (2012) meta-analysis all reported similar effect size ranges (*r* ≈0.40-0.50). This stability indicates that self-efficacy's predictive power for academic achievement is a highly robust psychological phenomenon that does not fluctuate substantially with changes in era, culture, educational level, or measurement instrument. This provides strong cumulative support for the core propositions of self-efficacy theory. By simultaneously modeling multiple ICT indicators, this study clarified the boundary conditions of the ICT paradox: the issue is not “whether to use technology” but “how to use technology.” General use frequency (school ICT, weekday duration) was negatively associated with achievement, while purpose-specific subject use was positively associated. This finding advances the ICT paradox from a simplistic “technology is harmful” interpretation to a refined understanding that “quality of use determines benefit,” providing a more operational framework for educational technology research and practice. Study time, teacher support, gender, and other variables are often viewed as important determinants of achievement in educational discussions. This study demonstrates that these variables have no independent predictive power after controlling for self-efficacy and socioeconomic background; their surface effects may primarily reflect covariation with psychological factors. This finding prompts researcher to reconsider variable selection and interpretation in educational research: many ostensibly important variables may be proxy indicators for deeper processes rather than independent causal factors. Japanese students' “high achievement–low self-efficacy” pattern was once viewed as a challenging case for cross-cultural applicability of Western motivation theories. This study demonstrates that despite below-average absolute self-efficacy levels, predictive power in the Japanese sample is fully consistent with Western research. This finding dispels concerns about “East Asian exceptionalism” and confirms the universality of social cognitive theory's core mechanisms across different cultural contexts.

This study has three implications for educational practice. The finding that self-efficacy is the strongest predictor suggests that educational interventions should prioritize building efficacy beliefs rather than simply increasing study time or technology equipment. Bandura (1997) identified four sources of self-efficacy: mastery experiences (successfully completing challenging tasks), vicarious experiences (observing peers succeed), verbal persuasion (encouragement from significant others), and physiological and emotional states (reducing anxiety). Teachers can enhance students' mathematics self-efficacy by designing appropriately challenging learning tasks, providing specific positive feedback, demonstrating peer success examples, and creating supportive classroom climates. The ICT paradox finding suggests that educational technology's benefit depends on quality of use, not quantity. Teacher training should focus on designing digital learning activities aligned with curriculum objectives rather than generically promoting ICT use frequency. When policymakers invest in educational technology infrastructure, they should simultaneously provide teacher professional development programs to ensure technology is purposefully integrated into instructional processes. “Hardware-first” strategies that provide equipment without pedagogical support may not produce expected learning benefits. The persistent SES influence suggests that educational equity policies need to go beyond hardware provision and opportunity expansion to address implicit gaps in cultural and psychological resources. Students from low-SES families may lack home environments conducive to self-efficacy building (such as parental academic expectations, educational involvement, and learning resources). Schools can address this gap by providing additional psychological support, mentoring systems, and intervention programs targeting efficacy beliefs.

Several patterns revealed in this study merit deeper exploration in subsequent research. This study employed a cross-sectional design and cannot establish causal direction between variables. Future research could employ longitudinal designs to track the bidirectional relationship between self-efficacy and achievement or employ experimental designs to test the causal effects of self-efficacy interventions on achievement. Structural equation modeling could simultaneously test multiple mediation pathways (such as teacher support → self-efficacy → achievement) to clarify indirect mechanisms of non-significant variables. ICT variable measurement in this study was relatively coarse, unable to distinguish different types of technology use (such as entertainment use, information retrieval, collaborative learning, subject-specific applications). Future research could develop more refined ICT use typologies to identify which types of technology use promote learning and which impede learning. Digital diary methods or application usage tracking could provide more objective behavioral data than self-report. PISA data have nested structure (students nested within schools), and standard regression cannot separate school-level from student-level effects. Future research should employ multilevel models to examine school-level variables such as school-average self-efficacy and school ICT policies. Marsh and Hau (2003) Big-Fish-Little-Pond Effect research demonstrated that school-level variables can have effects on student psychology opposite to individual-level effects, a complexity requiring multilevel modeling to capture. This study's results apply only to Japanese 15-year-olds. Future research could employ cross-national comparative designs to test cultural moderation of self-efficacy's predictive power. Sources, expression, and function of self-efficacy in East Asian collectivist cultures vs. Western individualist cultures may differ—differences worth systematic exploration.

### Limitations

This study has five limitations. First, the cross-sectional design cannot establish causal relationships: self-efficacy may be both a predictor and an outcome of achievement; the negative association between ICT use and achievement may reflect reverse causality (low achievers receiving more technology interventions). Second, ceiling effects in ICT measurement (school and home ICT use skewness both exceeded−3) limited variability and may have attenuated correlations; Japan as a highly technology-saturated society has inherently small individual differences in ICT accessibility. Third, standard regression did not account for PISA's nested data structure, potentially underestimating standard errors and inflating significance; future research should employ multilevel models to separate school effects. Fourth, although treating Likert scale data as continuous is conventional in PISA research and has methodological support (Lumley et al., 2002; Norman, 2010), this practice remains controversial (Sullivan and Artino, 2013) and requires cautious interpretation. Fifth, results apply only to Japanese 15-year-olds; generalization to other age groups, educational levels, or cultural contexts requires further validation.

## Conclusion

This study constructed a parsimonious six-variable model of mathematics achievement among Japanese adolescents using PISA 2022 data, explaining 35.4% of variance. The findings have important implications for motivational theory, educational technology research, and practice.

The core findings must be understood within the context of existing scholarship. Regarding the relationship between self-efficacy and academic achievement, Bandura (1997) social cognitive theory provided theoretical predictions, while Pajares and Miller (1994) and Richardson et al. (2012) validated these predictions in Western samples. However, the “high achievement–low self-efficacy” pattern commonly observed among East Asian students posed a challenge to the cross-cultural applicability of this theory. By replicating an effect size consistent with Western research in the Japanese sample, this study directly addresses this theoretical controversy: self-efficacy's predictive function does not depend on high absolute levels but rather derives from relative positioning within individual differences. This finding complements Marsh and Hau (2003) cross-cultural research on the Big-Fish-Little-Pond Effect, they demonstrated the cross-cultural consistency of social comparison processes, while the present study demonstrates the cross-cultural consistency of efficacy beliefs' predictive power.

Regarding ICT use and academic achievement, the existing literature presents an apparently contradictory picture. By simultaneously including multiple ICT dimensions in a single model, this study reveals that these seemingly contradictory findings can be integrated into a unified framework: general use frequency was negatively associated with achievement, while subject-specific use was positively associated. This pattern indicates that prior research discrepancies stem from measurement-level differences rather than substantive theoretical contradictions. “Purpose of use” rather than “frequency of use” is the key boundary condition determining technology's educational benefit. Regarding study time and achievement, the observation that study time lost significance after controlling for self-efficacy provides a mechanistic explanation for the “homework time paradox” proposed by Trautwein (2007) and Dettmers et al. (2009): the benefits of time investment may primarily operate through self-regulated learning processes rather than having independent direct effects.

This study advances theoretical development in three respects. First, by replicating the self-efficacy effect size in a Japanese sample, it provides cumulative support for the core propositions of efficacy theory from a different era, culture, and educational level. Second, by identifying the redundancy of “commonsense” variables, it prompts researchers to reconsider variable selection in educational research: many ostensibly important variables may be proxy indicators for deeper psychological processes rather than independent causal factors. Third, by simultaneously modeling multiple ICT indicators, it clarifies which ICT dimensions have independent predictive power, providing a more operational analytical framework for educational technology research.

The practical implications can be summarized in three recommendations. First, the focus of intervention should shift from external resource investment to psychological resource construction, educational reform should prioritize investment in cultivating students' efficacy beliefs rather than simply extending study time or expanding technology equipment. Second, technology integration strategies should shift from “increasing use” to “optimizing use” technology's educational benefit depends on quality rather than quantity, and “hardware-first” strategies may not only be ineffective but may even backfire. Third, educational equity policies should extend from equalizing opportunities to equalizing psychological resources, achieving educational equity requires attention to implicit gaps in self-efficacy construction and learning strategy mastery among low-SES students, not merely hardware provision and enrollment expansion.

The cross-sectional design of this study cannot establish causal relationships; future research could employ longitudinal designs to track bidirectional causality between self-efficacy and achievement. Standard regression did not separate school-level from student-level effects; multilevel models could address this limitation. Results apply only to Japanese 15-year-olds; cross-national comparisons could test the generalizability and cultural specificity of these findings. In summary, this study contributes to the field of motivational dynamics in technology-enhanced learning by providing confirmatory evidence for the cross-cultural applicability of social cognitive theory from a high-achieving East Asian context, advancing the crude debate of “whether technology is harmful” to a refined understanding of “which uses are harmful and which are beneficial,” and prompting researchers and policymakers to shift intervention focus from surface resource investment to deep psychological mechanisms. These findings converge on a core conclusion: in educational environments where technology is increasingly ubiquitous, psychological factors, especially domain-specific self-efficacy beliefs, remain the key determinants of academic success.

## Data Availability

The original contributions presented in the study are included in the article/Supplementary material, further inquiries can be directed to the corresponding author/s.
