# Supplementary material for: Motivational dynamics in technology-enhanced learning: a parsimonious model of self-efficacy, ICT use, and mathematics achievement
Source: Front Psychol. 2026 Feb 25;17:1745491. doi: 10.3389/fpsyg.2026.1745491 (PMC12977072; doi:10.3389/fpsyg.2026.1745491)
Supplement: Supplementary file 1 [file Data_Sheet_1.pdf]

Appendix

Appendix A: Data Collection Procedure

PISA 2022 data collection occurred during the 2022 school year via computer-based assessment. Students completed a 2-hour mathematics, reading, and science assessment followed by a 30–40-minute background questionnaire covering learning environments, attitudes, and socioeconomic background.

All procedures received ethical approval from participating countries' institutional review boards. Schools and students were selected using a two-stage stratified random sampling design. In the first stage, schools were sampled with probability proportional to the number of 15-year-old students enrolled. In the second stage, 42 students were randomly selected within each sampled school. Student participation was voluntary, with informed consent obtained from parents or guardians for students under 18.

The current study involved secondary analysis of de-identified, publicly available data accessed through the OECD PISA database (https://www.oecd.org/pisa/data/). All identifying information was removed prior to data release to ensure participant confidentiality.

Appendix B: Missing Data and Outlier Analysis

B.1 Missing Data Analysis

Independent samples t-tests were conducted to compare students with complete versus incomplete data on key variables. Results are presented in Table B1.

Table B1. Comparison of Students with Complete vs. Incomplete Data[8]

Variable	Complete Data (n = 5,438)	Incomplete Data (n = 322)	t	df	p	Cohen's d
Mathematics Achievement	M = 539.47, SD = 92.04	M = 469.45, SD = 98.25	12.47	355.17	< .001	0.76
Study Time	M = 5.01, SD = 2.95	M = 4.62, SD = 3.04	2.57	367.89	.010	0.13
Mathematics Self-Efficacy	M = -0.46, SD = 1.20	M = -0.69, SD = 1.28	3.42	369.12	.001	0.19
Socioeconomic Status (ESCS)	M = -0.00, SD = 0.71	M = -0.21, SD = 0.73	5.41	371.45	< .001	0.29

These patterns suggest that data were missing at random (MAR) rather than missing completely at random (MCAR), with missingness related to achievement-related variables. However, the high retention rate (94.4%) minimizes potential bias from listwise deletion.

B.2 Multivariate Outlier Detection

Multivariate outliers were identified using Mahalanobis distance (D^2), which measures the distance of each case from the centroid of all cases in multidimensional space. The critical value was determined using the chi-square distribution with degrees of freedom equal to the number of predictors:

Critical value: χ^2 (9, p = .999) = 27.88; Cases flagged: 201 (3.7% of sample); Criteria: Cases with $D^2 > 27.88$ were considered multivariate outliers

Given that multivariate outliers can substantially distort regression estimates, particularly coefficient standard errors and significance tests, these cases were excluded from analysis. Sensitivity analyses comparing results with and without outliers confirmed that their removal improved model stability without altering substantive conclusions.

Appendix C: Detailed Variable Definitions

This appendix provides detailed definitions and descriptive statistics for all variables included in the analysis, categorized for clarity.

Table C1. Variable Selection & Definition

Category	Variable name	PISA Code	Description	Measurement / Composition	Scale / Coding	Sample statistics (M, SD)	Sample range
Dependent variable	Math score	PV1MATH	The first plausible value of the PISA 2022 Mathematics Assessment	Mathematical literacy score, with an international average of ≈ 500 and SD ≈ 100	Score	541.32, 91.31	164-808 (Approximately Normal)
ICT-related variables	School ICT use	ICTSCH	A standardized index measuring the frequency of ICT use in school learning	Frequency of using digital devices for learning (Research, Projects, Exercises)	The higher the value, the higher the frequency	0.18, 0.65	Severe negative skewness (Ceiling effect)
	Home ICT Use	ICTHOME	A standardized index measuring the frequency of ICT use in school-related activities at home	Frequency of using digital devices at home for homework, studying, and exam preparation	The higher the value, the higher the frequency	0.26, 0.51	Severe negative skewness (Extreme ceiling effect)
	Subject-specific ICT use	ICTSUBJ	An index measuring the use of digital devices in mathematics, science, and language learning	Frequency of using digital devices in mathematics, science, and assessed language subjects	The higher the value, the higher the frequency	-0.02, 0.91	Approximately normal
	Weekday ICT use	ICTWKDY	A standardized index measuring the duration of ICT use on a typical workday	Typical weekday duration of using digital devices outside school (including entertainment, social media, and learning)	The higher the value, the longer the usage time	0.05, 0.98	Approximately normal
	ICT self-efficacy	ICTEFFIC	A standardized index measuring students' confidence in completing various ICT tasks	Confidence in completing digital device tasks (editing photos, creating presentations, finding information, coding)	The higher the value, the higher the confidence	-0.08, 0.97	Approximately normal
Learning-related variables	Learning time	STUDYHMW	Weekly hours spent on homework and studying outside school	Self-reported weekly hours	Hours	4.99, 2.96	0-10 hours (approximately normal)
	Mathematics self-efficacy	MATHEFF	A standardized index measuring confidence in completing mathematics tasks	Confidence in solving mathematics problems (calculating	International mean = 0, SD = 1	-0.47, 1.21	Approximately normal (Japanese students below the OECD average)

				discounts, interpreting graphs, solving equations)			
	Teacher support	TEACHSUP	A standardized index measuring perceived teacher support in mathematics classes	Frequency of teacher support in mathematics classes (caring, extra help, ensuring understanding)	International mean = 0, SD = 1	0.26, 0.99	Approximately normal
Control variable	Socioeconomic status	ESCS	PISA index of economic, social and cultural status	Parental education, parental occupational status (ISEI), family possessions (books, educational resources, cultural assets)	International mean = 0, SD = 1	-0.01, 0.71	Approximately normal (relatively homogeneous)
	Gender	FEMALE	A dichotomous variable indicating students' gender	0 = Male, 1 = Female	Dichotomous variable	N/A	51.1% Male, 48.9% Female

Appendix D: Regression Diagnostics

D.1 Multicollinearity Assessment

Variance Inflation Factors (VIF) were computed for all predictors to assess multicollinearity. VIF values indicate how much the variance of a regression coefficient is inflated due to collinearity with other predictors.

Table D1. Variance Inflation Factors[6]

Predictor	VIF
ICTSCH	1.18
ICTHOME	1.16
ICTSUBJ	1.08
ICTWKDY	1.11
ICTEFFIC	1.07
STUDYHMW	1.03
MATHEFF	1.09
TEACHSUP	1.04
ESCS	1.10
FEMALE	1.03

Interpretation: All VIF values were well below the conventional threshold of 10 (and even the more conservative threshold of 5), indicating no multicollinearity concerns.

D.2 Linearity and Homoscedasticity

Visual inspection of residual plots was conducted:

- Residuals vs. Fitted Values Plot: Showed no clear pattern, with residuals randomly scattered around zero, indicating linear relationships and homoscedastic residuals.
- Scale-Location Plot: Showed relatively constant spread of standardized residuals across fitted values, though slight heteroscedasticity was detected by the Breusch-Pagan test (BP = 26.24, p = .003). To address this, heteroscedasticity-consistent (HC3) robust standard errors were used for all inference.

D.3 Normality of Residuals

A Q-Q (quantile-quantile) plot was examined to assess normality of residuals:

- Finding: The Q-Q plot showed that residuals closely followed the theoretical normal distribution along most of the range, with minor deviations in the extreme tails (indicating slightly heavier tails than a perfect normal distribution).
- Interpretation: Given the large sample size (n = 5,237), the Central Limit Theorem ensures that regression estimates and hypothesis tests remain valid even with minor departures from normality.

D.4 Influential Cases

Influence diagnostics were conducted using Cook's distance (D) and leverage values:

- Cook's Distance: Measures the influence of each case on regression estimates. No cases exceeded the threshold of $D > 1$, indicating no extreme influential outliers.
- High Leverage Cases: Approximately 12.6% of cases showed relatively high leverage (exceeding $2(k + 1)/n$, where k = number of predictors). However, these cases did not have high Cook's D values, suggesting they did not disproportionately influence results.
- Sensitivity Analysis: The regression model was re-estimated after excluding high-leverage cases. Results were substantively identical (coefficient changes < 0.02, all significance patterns unchanged), confirming model robustness.

D.5 Independence of Errors

Given the nested structure of PISA data (students within schools), potential violations of independence were considered:

- Design Effect: PISA employs complex sampling with clustering. However, the current study focuses on individual-level predictors and does not model school-level effects.
- Intraclass Correlation: Prior PISA research indicates that school-level variance in mathematics achievement is relatively modest in Japan compared to other countries, reducing concerns about dependency.
- Note: A limitation of this study is that standard regression does not account for clustering. Future research should employ multilevel modeling to properly partition within-school and between-school variance.

Appendix E: Technical Implementation Notes

Several advanced statistical procedures required in this study are not available in JAMOVI's standard interface or require manual implementation. These were executed using JAMOVI's Rj module with appropriate R packages, or through manual procedures within JAMOVI's interface. Table E1 summarizes all technical implementations.

Table E1. Summary of Technical Implementations Beyond JAMOVI's Standard Features

Procedure	Issue	Implementation Method	R Packages Used
HC3 Robust Standard Errors(Hayes & Cai, 2007; Zeileis, 2004)	Not available in standard linear regression module	JAMOVI Rj module with R code	sandwich, lmtest
Breusch-Pagan Test	Requires external module installation	JAMOVI moretests module (Koenker version)	N/A (JAMOVI module)

Backward Elimination	No automated algorithm in JAMOVI	Manual iterative model comparisons	N/A (JAMOVI module)
Mahalanobis Distance	Not available for outlier detection	JAMOVI Rj module with R base functions	R base stats package
Little's MCAR Test	Not available in JAMOVI	Manual independent samples t-tests comparing complete vs. incomplete cases	N/A (JAMOVI module)
Cook's Distance Extraction	Visual display only, no case filtering	JAMOVI Rj module for extracting diagnostic values	R base stats package
VIF Detailed Output	Standard output available but formatting customized	AMOVI Assumption Checks + custom R formatting	N/A (JAMOVI module)
Nested Model F-test	Not directly provided in model comparison	Manual calculation or R anova() function via Rj module	R base stats package
